# CD100 Effects in Macrophages and Its Roles in Atherosclerosis

**DOI:** 10.3389/fcvm.2018.00136

**Published:** 2018-09-28

**Authors:** Maria C. A. Luque, Mariana K. Galuppo, Janaina Capelli-Peixoto, Beatriz S. Stolf

**Affiliations:** ^1^Heart Institute, Universidade de São Paulo, São Paulo, Brazil; ^2^Parasitology, Universidade de São Paulo, São Paulo, Brazil

**Keywords:** macrophages, atherosclerosis, cell recruitment and activation, angiogenesis, CD100/SEMA4D

## Abstract

CD100 or Sema4D is a protein from the semaphorin family with important roles in the vascular, nervous and immune systems. It may be found as a membrane bound dimer or as a soluble molecule originated by proteolytic cleavage. Produced by the majority of hematopoietic cells including B and T lymphocytes, natural killer and myeloid cells, as well as endothelial cells, CD100 exerts its actions by binding to different receptors depending on the cell type and on the organism. Cell-to-cell adhesion, angiogenesis, phagocytosis, T cell priming, and antibody production are examples of the many functions of this molecule. Of note, high CD100 serum levels has been found in inflammatory as well as in infectious diseases, but the roles of the protein in the pathogenesis of these diseases has still to be clarified. Macrophages are highly heterogeneous cells present in almost all tissues, which may change their functions in response to microenvironmental conditions. They are key players in the innate and adaptive immune responses and have decisive roles in sterile conditions but also in several diseases such as atherosclerosis, autoimmunity, tumorigenesis, and antitumor responses, among others. Although it is known that macrophages express CD100 and its receptors, few studies have focused on the role of this semaphorin in this cell type or in macrophage-associated diseases. The aim of this review is to critically revise the available data about CD100 and atherosclerosis, with special emphasis on its roles in macrophages and monocytes. We will also describe the few available data on treatments with anti-CD100 antibodies in different diseases. We hope that this review stimulates future studies on the effects of such an important molecule in a cell type with decisive roles in inflammatory diseases such as atherosclerosis.

## Atherosclerosis and semaphorins

Atherosclerosis is one of the most prevalent diseases in the world and the main cause of myocardial infarction and stroke (1). It is a multistage chronic vascular inflammatory disease that develops at the inner curvatures and branch points of medium and large arteries, usually associated with a disturbed blood flow (2). Progressive accumulation of low density lipoprotein (LDL), fibrosis, and inflammation within the subendothelial space give rise to fatty streaks or fatty plaques, which results in the thickening of the intima layer of the arteries. Vascular inflammation is triggered by a collection of infiltrating inflammatory cells such as monocytes, macrophages, T lymphocytes and dendritic cells, in addition to vascular smooth muscle cells (VSMCs), extracellular matrix (ECM) proteins, lipids, and calcium deposits (3). The persistence of inflammatory myeloid-derived cells, especially macrophages, is common in atherosclerotic plaques (4). These cells are involved in lesion progression and plaque instability through the secretion of extracellular-degrading proteases and cytotoxic factors (4).

One of the first steps in atherogenesis is endothelial cell (EC) dysfunction and activation, which is initiated by oxidized low density lipoprotein (oxLDL), proinflammatory cytokines, oxidative stress, hypertension, hyperglycemia, aging, and shear stress (5, 6). This condition is also marked by an imbalance between vasorelaxation induced by nitric oxide and prostacyclin and vasoconstriction induced by endogenous agents such as endothelin-1 (5). Alterations of EC permeability, transport, and transcytosis lead to LDL accumulation in the intima layer, which becomes trapped to ECM proteins (7). There is also an increase in the production of reactive oxygen species (ROS), which further oxidize LDL to ox-LDL (5). All these factors lead to an increase in the expression of EC adhesion molecules such as vascular cell adhesion molecule-1, intercellular adhesion molecule 1, and selectins, which interact with monocyte and T lymphocyte surface molecules, inducing their arrest and transmigration into the arterial wall (5, 6). Monocytes are so important that for many years, the development and progression of atherosclerotic plaques have been viewed as a classical monocyte driven process (8, 9). Indeed, monocytes differentiate into macrophages and foam cells, which are the most abundant cell types in established lesions, although T cells dominate in early stages (10). Cholesterol activates the inflammasome in macrophages, leading to the secretion of active IL-1β and IL-18, which induce the release of ROS and matrix degrading enzymes, and the activation of T cells (7). Besides, activated macrophages produce ROS and are capable of up taking vast amounts of oxLDLs, originating foam cells (11). Pro-inflammatory macrophages and foam cell death within the fatty streak propagate inflammation and may induce angiogenesis, perpetuating plaque development (12).

The progression of fatty streaks to fibrous atherosclerotic plaques depends on the proliferation of VSMCs, which accumulate in the intima and produce ECM. On the other hand, vascular inflammation causes degradation of this ECM, reducing fibrous cap formation, and generating less fibrous plaques (5). The subsequent activation of the coagulation cascade causes intravascular thrombus formation and may lead to acute clinical events such as stroke (5). Besides the molecules traditionally described as implicated in atherogenesis and lesion progression, an increasing number of studies have shown the roles of microRNAs (miRNAs) in processes that are decisive in atherosclerotic lesion formation [reviewed in (13)]. In fact, miRNAs regulate pathophysiological processes and signaling pathways in endothelial cells (ECs), VSMCs, and macrophages, and affect lipid homeostasis. These miRNAs are induced in response to biomechanical or biochemical stimuli. More recently, long non-coding RNAs have also been implicated in several pro-atherosclerotic processes (13).

Semaphorins are among the molecules that can either favor or inhibit cell recruitment to atherosclerotic plaques. The semaphorin family includes more than 20 highly conserved proteins which may be secreted, transmembrane, or glycosylphosphatidylinositol-linked, all of which have a homologous extracellular Sema domain with 500 amino acids (14). They have been identified in species from invertebrates to humans and virus (14) and are grouped into eight subclasses (15). Semaphorins were first described in the nervous system, and they play important roles in processes such as migration, proliferation and cellular aggregation, cytoskeletal rearrangement, neuronal development and synaptic transmission, angiogenesis and cardiovascular development, and immune responses (14–18). Signal transduction occurs through binding to receptors such as neuropilin 1, plexins (plexins from classes A and B, plexin C1, and plexin D1), CD72, Tim-2, integrins, TREM2, and DAP12 (15, 19).

**Figure 1:**
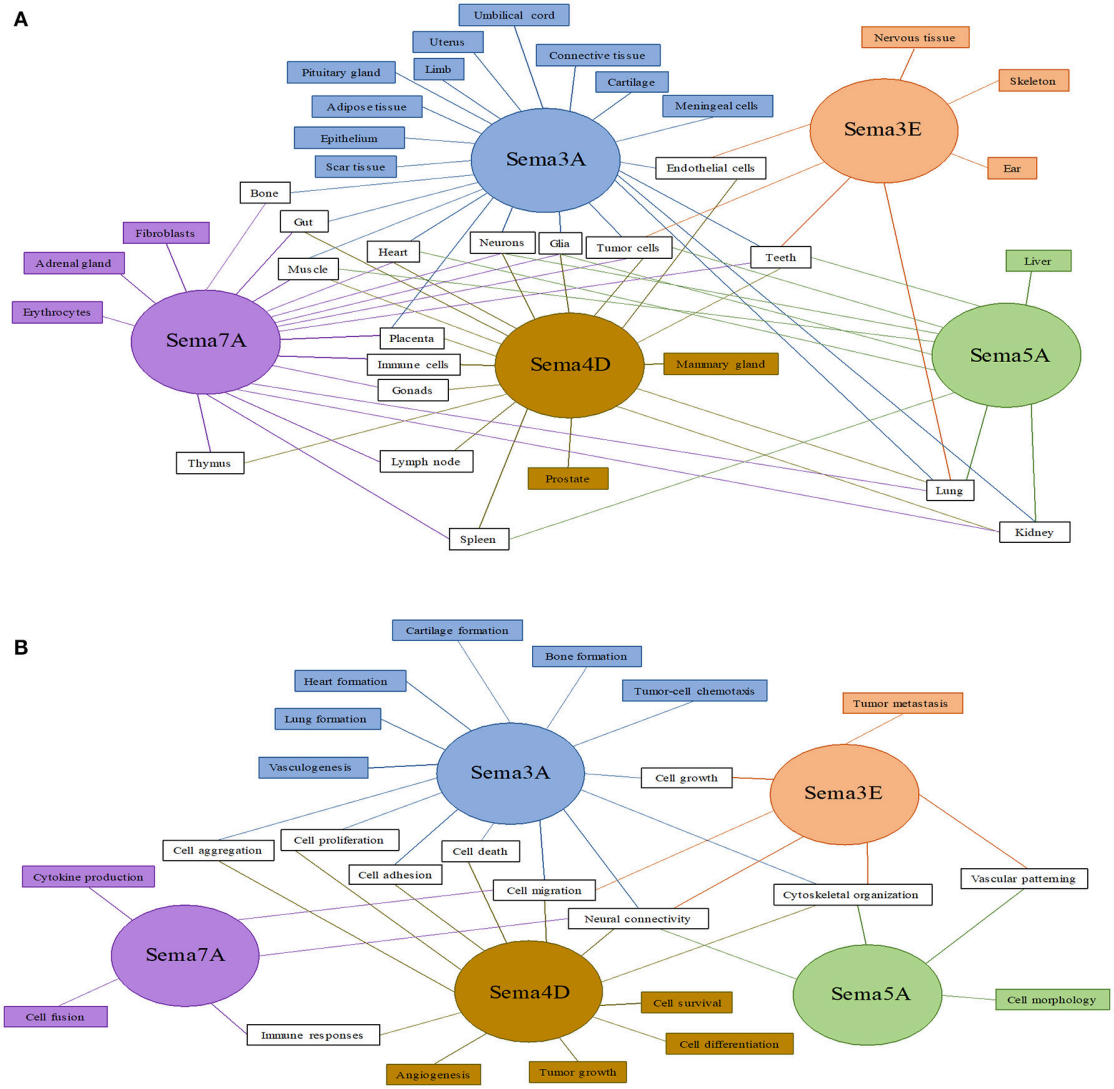
Expression and function of semaphorins 3A, 3E, 4D, 5A, and 7A, which are important in cardiovascular diseases. Expression of these semaphorins in different tissues and cells **(A)** and their functions in different biological processes **(B)**. Sites and funtions exclusive for one semaphorin are shown in rectangles of the corresponding color, sites and functions shared by two or more semaphorins are depicted in white rectangles. Adapted from Yazdani and Terman (14).

Several semaphorins are known to be involved in cardiovascular diseases, mainly Sema 3A, 3E, 4D, 5A, and 7A. The tissues and cells in which these five semaphorins have already been detected are summarized in Figure 1A, and the several functions reported for each of them are shown in Figure 1B.

These five semaphorins have been implicated in different processes associated with atherosclerosis and ischemia (20). Sema 3A is expressed by endothelial cells and inhibits monocyte migration. Pro-atherogenic conditions such as oscillatory blood flow and pro-inflammatory cytokines reduce Sema3A expression and increase adhesion of monocytes to endothelia (21). Similarly, Sema3E binding to plexin D1 reduces macrophage migration. This semaphorin is highly expressed in inflammatory M1 macrophages, and is downregulated in M2 macrophages, the subtype that predominates in regressing plaques (22). Sema3E also inhibits smooth muscle cell migration and proliferation, decreasing neointimal hyperplasia in animal model of atherosclerosis (23). Different from Sema3A and 3E, Sema7A favors atherosclerotic plaque development by increasing endothelial dysfunction in a β1 integrin-dependent manner (24). In fact, vessels under oscillatory disturbed shear stress show higher Sema7A levels, while Sema7A knockout (ko) mice have attenuated plaque formation (24).

Although Sema4D (also called CD100) is expressed in platelets, monocytes, macrophages and T cells, cell types that are pivotal in atherosclerosis, only a few studies have explored the roles of this semaphorin in the disease. The focus of this review is to critically compile the available data on the role of this molecule in atherosclerotic plaque development and progression.

## Main roles of CD100 in different cell types

Semaphorin 4D, Sema4D, or CD100 (in this review referred to as CD100) belongs to semaphorin class 4 and is one of the so called “immune semaphorins” (25). It is known to be involved in the pathogenesis of several diseases such as atherosclerosis and multiple sclerosis (26), rheumatoid arthritis (27), encephalomyelitis (28), multiple myeloma (29), ANCA-associated vasculitis (30), systemic lupus erythematosus (31), and many other tumors (26). The inflammatory and pro-angiogenic components common to most of these diseases points to the important role of the molecule in the immune system and in the function of endothelial cells. Indeed, CD100 is expressed by most cells of the hematopoietic system, including lymphoid cells such as B and T lymphocytes and natural killer cells, as well as myeloid cells (neutrophils, platelets, and monocytes) and endothelial cells, and its expression usually increases upon activation (32, 33). T cells exhibit high levels of CD100 (34, 35), probably followed by neutrophils (30), platelets (36), and monocytes (37, 38).

In addition to being expressed in the membrane, CD100 is also found in a soluble form (sCD100), generated from membrane CD100 (mCD100) by proteolytic cleavage (39, 40) in an activation-dependent manner (41). Activated T and B cells are known to shed sCD100, and mice immunized with T-cell–dependent antigens or MRL/lpr mice with autoimune disease have increased sCD100 serum levels (31). Platelets also shed sCD100 as thrombus formation progresses (36), and macrophages cleave the membrane-bound molecule upon STING (“stimulator of interferon genes”) activation with agonists (42).

Both membrane and soluble CD100 have functional properties. mCD100 is involved in the interaction between different cell types. It mediates platelet-platelet and platelet EC contact, favoring thrombus formation (36); it also increases monocyte-endothelial cell interaction (both of which express CD100 and its receptors). Other examples include mCD100 participation in DC-T cell interaction increasing activation, proliferation and differentiation of T cells (31, 41, 43), as well as inducing DC maturation (31). Macrophages are also recruited to renal glomeruli in experimental glomerulonephritis based on mCD100-plexin B1 interactions. Curiously, binding of neutrophils' CD100 to endothelial cells leads to a decrease in activation of the first cells and reduced net formation (30).

The roles of sCD100 were also analyzed in several systems. The soluble molecule shed by macrophages promotes tumor angiogenesis (44), and sCD100 released by platelets in thrombus may interact with endothelial cells, monocytes and other platelets (36). In the immune system, sCD100 increases proliferation, differentiation, and IgG1 production by stimulated mouse B cells (45, 46). In monocytes and DCs, it increases IL-10 secretion and reduces IL-6, IL-8, and TNF-α (47). Besides, it inhibits migration of human B cells (48), monocytes, and immature DCs (47). We have recently shown that sCD100 affects oxLDL phagocytosis by human macrophages (32) and *Leishmania* internalization by mouse macrophages (49).

## Receptors for CD100: plexins and CD72

Both membrane bound CD100 dimer and sCD100 interact with specific receptors, resulting in distinct biologic activities. CD100 receptors are heterogeneously expressed depending on the cell type and organism. In humans, plexin B1 is expressed in a broad range of non-immune cells, and functions as the high affinity receptor for CD100 (50), having decisive roles in angiogenesis and in vascular diseases (32, 36, 44, 51). In the immune system plexin B1 is expressed only in follicular dendritic cells, bone marrow stromal cells and (at lower levels) in activated T cells, but not in monocytes, macrophages, other dendritic cells and quiescent T and B lymphocytes (52, 53). In mice, plexin B2 has been described in germinal center B cells (54), in epithelial γδ T cells (55) and in macrophages, conventional and plasmacytoid dendritic cells (56). In humans, plexin B2 cDNA has been found in myeloid cells (56). More recently, plexin B2 has also been identified as a putative CD100 receptor in human monocytes, macrophages and foam cells (32). CD72 is considered the major receptor for CD100 in immune cells (41), and was shown to mediate CD100 effects in murine macrophages (57), in bronchial epithelial cells, B cells, dendritic cells, fibroblasts, mast cells, and basophils (41, 52, 58, 59). Good reviews have compiled information on the structure of CD100 and its receptors plexin B1 and CD72 (30, 41, 43, 60, 61). For detailed structures, please refer to Kumanogoh and Kikutani (60).

## CD100 in atherosclerosis

The importance of CD100 in atherogenesis can be evidenced by the presence of both CD100 and its receptors in cell types which have been demonstrated to play crucial roles in the establishment of atherosclerotic lesions. As other semaphorins, CD100 also mediates cell-to-cell communication and adhesion in different contexts, such as in platelet-platelet interaction and in platelet and monocyte adhesion to endothelial cells, all of which will be detailed later. CD100 also affects cell activation and cytokine production, which may influence polarization of macrophages and lymphocytes.

CD100 roles in atherosclerosis were evaluated both in LDL^−/−^ and apolipoprotein E deficient (ApoE^−/−^) mice models, as well as in an injury model of thrombus formation. CD100 was shown to be involved in platelet-endothelial cell interaction, an important step in thrombus formation (36, 62). Platelets were shown to express CD100 and both CD72 and plexin B1, and cell surface levels of CD100 and CD72 increase during platelet activation (36). The absence of CD100 impaired platelet responses *in vitro* and *in vivo*, indicating that CD100 is involved in platelet hyperactivity. Moreover, mice lacking CD100 showed delayed and smaller arterial occlusion after vascular injury *in vivo* (36). In the dyslipidemic LDL receptor negative (LDLR^−/−^) mice model, platelet accumulation and thrombus formation were significantly reduced in CD100^−/−^ LDLR^−/−^ mice, resulting in less arterial occlusion when compared to LDLR^−/−^ mice (62). Besides, the absence of CD100 in these mice reduced lipid deposition and lesion size and decreased the frequency of arterial occlusion after feeding on a high fat diet (62). Further characterization of the double CD100^−/−^ ApoE^−/−^ mice model also evidenced the importance of CD100 in atherogenesis (63). The lack of CD100 in ApoE-deficient mice was found to slow the progression of atherosclerosis, resulting in decreased lipid staining, macrophage infiltration and intimal neovascularization in the aortic plaques (63). Together, these results suggest that CD100 acts as an enhancer of pathologic platelet activation and atherogenesis.

Our group demonstrated CD100 expression in human atheromas, more specifically in plaque macrophages and foam cells (38). *In vitro* cultured monocytes (34) and differentiated macrophages and foam cells were shown to express CD100 (38). Furthermore, sCD100 reduced oxLDL incorporation by macrophages by decreasing CD36 expression, pointing to a specific anti-atherogenic effect of the molecule (32).

The coupling of CD100 in endothelial cells to its receptor plexin B2 expressed in human monocytes, macrophages, and foam cells was also suggested as an important step for adhesion between monocytes and endothelial cells (32). In fact, we have shown that blocking of plexin B1 and B2 reduced monocyte binding to activated endothelial cells *in vitro*. This work proposed CD100 as a part of the wide range of adhesion molecules that promote cell-to-cell contact in monocyte-endothelial cell interaction, mediating the arrest, and migration of blood monocytes into the subendothelial space, showing another important role for CD100 in atherogenesis (32). Accordingly, it has recently been shown that macrophages display preferential adhering and spreading to a CD100-rich surface compared to a heparin coated surface (64).

On the other hand, sCD100 that is shed from activated macrophages in the tumor microenvironment induces angiogenesis (44). Although to this date the effect of sCD100 shed by macrophages in neoangiogenesis inside atherosclerotic plaques has not been addressed, we believe this may be another important outcome of this molecule in plaque development. This hypothesis is strengthened by the fact that, as already mentioned, the double KO mice for CD100 and ApoE presented less neovascularization in aortic plaques, what can be also true in human disease (63). Two independent studies of human cardiovascular diseases have described increased levels of sCD100 in the serum of patients with heart failure compared with healthy controls (65, 66), especially in those with diabetes (66). Soluble CD100 levels showed significant correlation with the levels of creatinine and brain natriuretic peptide, and rapidly decreased after clinical improvement (65). These patients also exhibited a larger population of CD100high T cells, suggesting that these cells may be involved in heart failure (66). Elevated serum levels of sCD100 were also described in patients with atrial fibrillation, the most common type of arrhythmia (67). These conditions are associated with inflammation and predispose to coagulation and thrombus formation, both of which are known to be influenced by CD100 (67).

Another effect, probably equally important, is that sCD100 may eventually alter macrophage differentiation and phenotype. As previously mentioned, human monocytes stimulated with sCD100 secrete higher levels of IL-10 and lower amounts of the proinflammatory cytokines IL-6, IL-8, and TNF-α (47). Accordingly, correlations were described between CD100 expression and M2 macrophage abundance in human ovary epithelial tumors and in the patients' ascites (68). Besides, the same study showed that monocytes stimulated with sCD100 expressed higher levels of CD163, suggesting a polarization toward the M2-like phenotype (68). Both M1 and M2 macrophage populations increase during atherogenesis, and the balance between them is dynamic over time. In fact, M1 macrophages predominate in situations of plaque progression while M2 are increased during the regression phase (69). Accordingly, M1 cells predominate in rupture-prone shoulder regions of human plaques, whereas M2 are more abundant in the adventitia and in stable areas of the plaques (69). The role of M2 macrophages in the resolution of inflammation in regressing plaques is probably due to the secretion of IL-10. Besides, these cells secrete collagen and are capable of efferocytosis, promoting remodeling of the area and clearance of apoptotic cells (69). It is still unknown whether sCD100 induces IL-10 secretion and reduces proinflammatory cytokine production by plaque macrophages, as previously shown for human blood monocytes (47). If so, CD100 would probably increase M2 cells and decrease M1. Since inflammatory monocytes and M1 macrophages show proatherogenic effector activities (12), it is possible that the reduction of these cells might diminish plaque formation.

**Figure 2:**
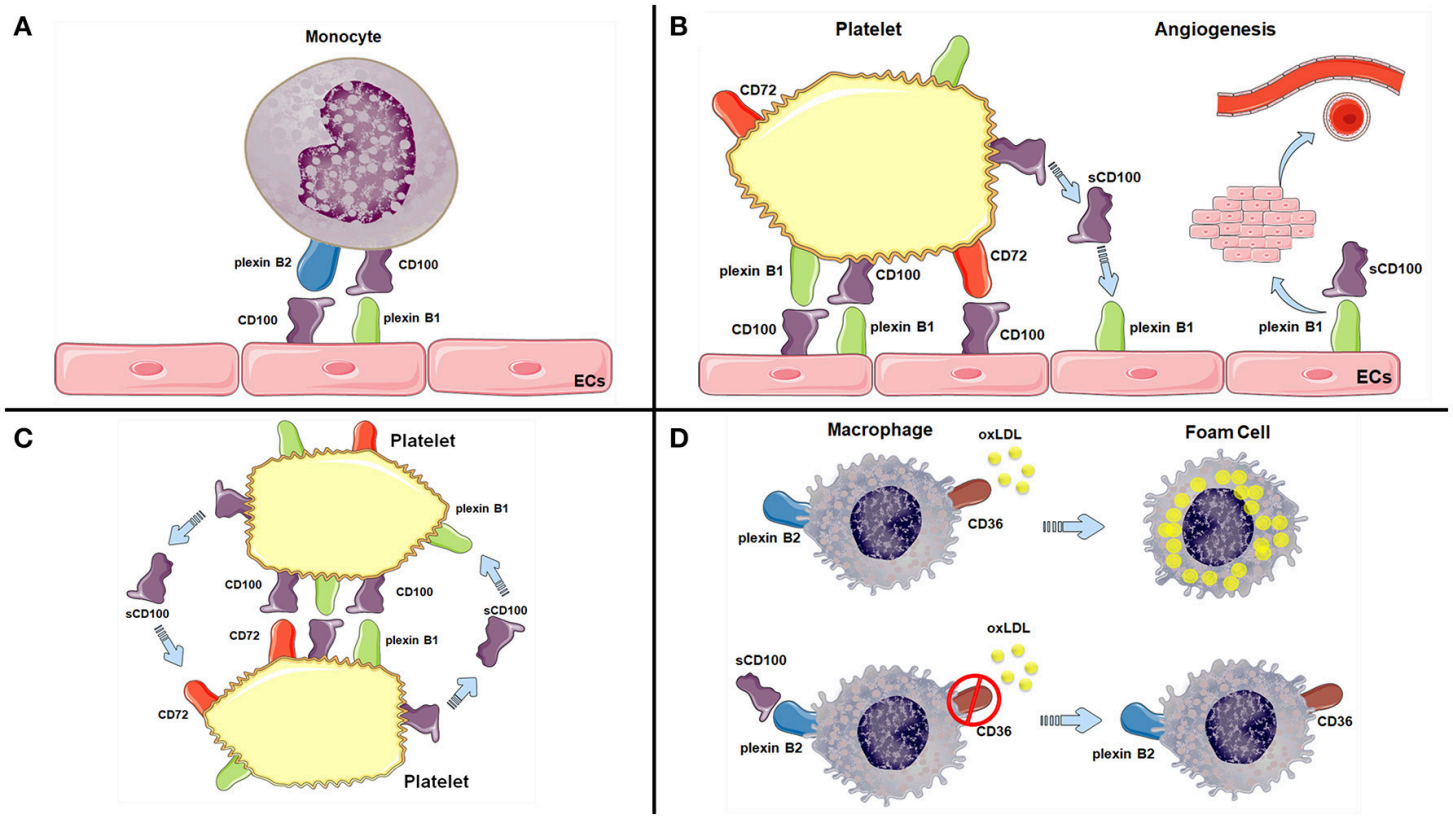
CD100 is involved in many important steps of atherogenesis. CD100 binding to plexin B2 at the cell surface monocytes and to plexin B1 on endothelial cells (ECs) promotes cell adhesion **(A)** and may facilitate monocyte transmigration. Platelets express CD100 and receptors CD72 and plexin B1, which are directly involved in platelet binding to ECs **(B)** and in platelet aggregation **(C)** and thrombus formation. sCD100 induces angiogenesis by binding to plexin B1 in ECs **(B)**. However, CD100 also presents atheroprotective roles, such as the reduction of oxLDL capture by macrophages through downregulation of scavenger receptor CD36 **(D)**. References as cited in Table 1.

Altogether, the studies mentioned in this review provide mechanistic links between CD100 and atherosclerosis development. Table 1 and Figure 2 summarize the potential effects described for CD100 in atherosclerosis. Proatherogenic roles of CD100 include monocyte and platelet recruitment to endothelial cells and induction of angiogenesis, while atheroprotective roles include reduction of oxLDL capture by macrophages and polarization of monocytes and macrophages to less inflammatory phenotypes. The complexity of atherogenesis and the contribution of different cell types to lesion progression may determine the balance of CD100 effects in each phase of the process and in patients individually.

**Table 1:** Effects of mCD100 and sCD100 in processes that may influence atherogenesis.

**Cells involved**	**CD100 receptor**	**Effect**	**Pro or antiatherogenic**	**References**
EC and monocyte	Plexin B1 and plexin B2	Increase in adhesion	Proatherogenic	(32)
Platelet-platelet and platelet- EC	Plexin B1 and CD72	Thrombus formation	Proatherogenic	(36, 62)
Endothelial cells	Plexin B1	Angiogenesis	Proatherogenic	(51, 63)
Macrophage	Plexin B2	Decrease in oxLDL internalization	Anti-atherogenic	(38)

*The table includes both mouse and human receptors for CD100 in each cell type*.

## Therapeutic strategies using anti-CD100: potential applications in atherosclerosis?

CD100 has been recently evaluated as a target for therapeutics of inflammatory diseases and cancer. In a rheumatic arthritis mouse model (collagen-induced arthritis), disease scores were significantly lower in anti-CD100 treated mice (27). Indeed, treated mice showed reduction in inflammatory infiltration into the synovium, erosion of cartilage and bone, as well as lower TNF-α and IL-6 serum levels. Besides, these mice had less angiogenesis at sites of inflammation (27), in agreement with CD100 pro-angiogenic role. A beneficial role for anti-CD100 treatment was also reported in a mouse model of colon cancer (70). While untreated mice displayed intense CD100 expression at the invasive margins of growing tumors, tumors of anti-CD100 treated mice did not show this CD100 gradient and had intense recruitment of activated monocytes and lymphocytes (especially of CD8+ T cells) into the tumor. Treated mice developed smaller tumors and had higher survival rates, especially when anti-CD100 was associated to other immodumodulatory therapies (70).

A humanized IgG4 anti-CD100 antibody (named VX15/2503) was recently developed using a hybridoma that recognizes the murine, primate, and human CD100 molecules (71). Phase I clinical trial employed this humanized antibody in 42 patients with advanced solid tumors (72). The treatment was well-tolerated, and 19% of the patients exhibited stable disease for at least 4 months (72). More recently, the same antibody was employed in a randomized, double-blind study involving patients with multiple sclerosis (73). The treatment was again well-tolerated, few patients reported treatment–related adverse events and all of them completed the study (73).

The positive results obtained with CD100 neutralization in the inflammatory mouse arthritis model, as well as the tolerability of the humanized anti-CD100 antibody suggest that anti-CD100 treatment could be explored as a therapeutic strategy for inflammatory diseases in which CD100 plays an important role, such as atherosclerosis.

## Author contributions

The structure of this review was designed by BS and modified by ML, JC-P, and MG. The text was writen mainly by BS, ML and JC-P.

### Conflict of interest statement

The authors declare that the research was conducted in the absence of any commercial or financial relationships that could be construed as a potential conflict of interest.
